# PACAP Alleviates Paclitaxel‐Induced Peripheral Neuropathy by Targeting Oxidative Stress and Mitochondrial Damage via the PGC‐1α Pathway

**DOI:** 10.1002/cns.70745

**Published:** 2026-01-22

**Authors:** Ruyue Mo, Chuanming Wang, Haibei Hu, Shuqi Shi, Di Cao, Zhenhui Luo, Hua Yang, Mingzhu Zhai, Wuping Sun

**Affiliations:** ^1^ Department of Clinical Laboratory Medicine, Fifth Affiliated Hospital Southern Medical University Guangzhou China; ^2^ Department of Pain Medicine Shenzhen Nanshan People's Hospital and the 6th Affiliated Hospital of Shenzhen University Medical School Shenzhen China; ^3^ Center for Medical Experiments (CME) Shenzhen Guangming District People's Hospital Shenzhen China; ^4^ School of Medical Technology North Minzu University Yinchuan China

**Keywords:** mitochondrial damage, oxidative stress, paclitaxel, pain, pituitary adenylate cyclase‐activating peptide

## Abstract

**Background:**

Paclitaxel‐induced peripheral neuropathy (PIPN) is a severe and dose‐limiting side effect. This study investigated the therapeutic potential of Pituitary Adenylate Cyclase‐Activating Polypeptide (PACAP) and its underlying mechanism.

**Methods:**

A murine PIPN model was established. Behavioral tests assessed neuropathic pain. Molecular and cellular analyzes, including western blot, ELISA, and transmission electron microscopy, evaluated oxidative stress, mitochondrial function, and key protein expression in dorsal root ganglia (DRG) and SH‐SY5Y cells. The PGC‐1α inhibitor SR‐18292 was used for mechanistic validation.

**Results:**

High‐dose PACAP (100 μg/kg) significantly alleviated PTX‐induced mechanical allodynia and thermal/cold hyperalgesia. It reduced oxidative stress (lowered ROS/MDA, increased SOD) and restored mitochondrial function (improved membrane potential, ATP, and ultrastructure) in DRG neurons. PACAP upregulated PGC‐1α and HO‐1 expression, and its protective effects were abolished by PGC‐1α inhibition. Crucially, PACAP did not interfere with PTX's antitumor efficacy.

**Conclusion:**

PACAP alleviates PIPN by activating the PGC‐1α pathway to improve mitochondrial function and counteract oxidative stress, presenting a promising adjunct therapy that does not compromise chemotherapy.

## Graphical abstract

This study demonstrates that PACAP alleviates paclitaxel‐induced peripheral neuropathy in mice. Its protective mechanism involves activating the PGC‐1α pathway to restore mitochondrial function and reduce oxidative stress in sensory neurons. Crucially, PACAP does not interfere with the anti‐tumor efficacy of paclitaxel.

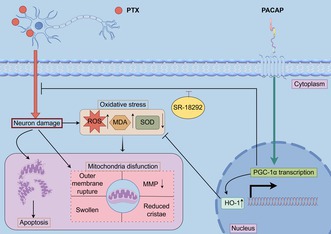

## 1 Introduction

The global incidence and mortality rates of cancer continue to rise persistently [1]. Chemotherapy, an important approach in cancer treatment, occupies a significant position within the therapeutic landscape. Paclitaxel (PTX), a widely used chemotherapeutic agent, is commonly administered for various malignancies, including breast cancer, ovarian cancer, and non‐small cell lung cancer [2, 3, 4]. However, the clinical use of PTX is frequently accompanied by severe adverse effects. Among these, PTX‐induced peripheral neuropathy (PIPN) is particularly prominent and represents one of its dose‐limiting toxicities.

PIPN primarily manifests as sensory symptoms, including numbness and tingling in the fingers and toes, sensory impairment triggered by cold stimuli, and neuropathic pain. These symptoms not only severely compromise patients' quality of life but may also lead to interruptions or delays in the treatment regimen [5, 6]. Current management strategies—such as gabapentin and pregabalin—provide suboptimal relief and are associated with adverse effects including drowsiness and dizziness [7]. Consequently, elucidating PIPN pathogenesis and developing novel therapeutic approaches are critical unmet needs.

Pituitary adenylate cyclase‐activating polypeptide (PACAP), a multifunctional neuropeptide widely distributed in the central and peripheral nervous systems, has emerged as a promising neuroprotective agent. Beyond its roles in neurodevelopment and cell survival [8, 9], PACAP demonstrates significant analgesic properties across diverse preclinical pain models [10, 11]. Studies indicate its ability to attenuate nociceptive behaviors in inflammatory [12], neuropathic [13], and migraine‐related pain contexts [14], likely through modulation of glial activity, neurotransmitter release, and downstream anti‐inflammatory pathways. Critically, substantial evidence highlights the efficacy of PACAP in mitigating oxidative stress [15], a key pathological driver in neurological disorders. This antioxidant capability, coupled with its analgesic potential, positions PACAP as a compelling candidate for PIPN intervention.

Peroxisome proliferator‐activated receptor‐γ coactivator‐1α (PGC‐1α) acts as a core regulator of mitochondrial biogenesis. It promotes mitochondrial generation and maintains their normal function by activating a series of transcription factors. Additionally, PGC‐1α regulates the expression of antioxidant enzymes, thereby helping cells resist damage caused by oxidative stress [16, 17]. Given PACAP's documented analgesic and antioxidant effects, we hypothesize that it may alleviate PIPN through PGC‐1α‐dependent mechanisms to restore mitochondrial homeostasis and redox balance.

In this study, we established in vitro and in vivo PIPN models to systematically evaluate the analgesic efficacy of PACAP using behavioral assessments. Concurrently, we analyzed oxidative stress markers and mitochondrial function parameters to elucidate the specific mechanisms by which PACAP mitigates PTX‐induced neuropathic pain. This study aims to provide a theoretical foundation and experimental support for developing novel treatment strategies for PTX‐induced PIPN, thereby improving the quality of life for cancer patients undergoing chemotherapy and enhancing the clinical utility of PTX.

## 2 Materials and Methods

### 2.1 Animals

C57BL/6J mice (male and female), aged 7 weeks and weighing 18–20 g, were obtained from the Guangdong Medical Laboratory Animal Center (Animal Production License No. SCXK (Yue) 2022‐0002). Mice were housed under controlled environmental conditions: temperature maintained at 22°C–24°C, relative humidity at 40%–60%, and a 12‐h light/dark cycle. Food and water were provided ad libitum. Following a one‐week acclimatization period, mice were employed in subsequent experimental procedures. All animal experiments strictly adhered to relevant ethical regulations and were approved by the Institutional Animal Care and Use Committee (IACUC) of Shenzhen Lingfutuopu Biotechnology Co. Ltd. (Ethics Approval No. TOP‐IACUC‐2024‐0106). Mice were randomly assigned to one of four experimental groups: (1) Vehicle group, (2) PTX group, (3) PTX + PACAP (30 μg/kg) group, and (4) PTX + PACAP (100 μg/kg) group.

### 2.2 Paclitaxel‐Induced Peripheral Neuropathy

To establish the PIPN model, mice received intraperitoneal (i.p.) injections of PTX (i.p., 4 mg/kg per injection) on days 0, 2, 4, 6, 8, 10, 12, and 14, yielding a cumulative dose of 32 mg/kg as previously reported [18, 19, 20]. Additionally, PACAP was administered via injection (i.p.) on days 1, 3, 5, 7, 9, 11, 13, 15, 17, and 19 at doses of 30 μg/kg or 100 μg/kg per injection, resulting in cumulative doses of 300 μg/kg and 1 mg/kg, respectively following previous reports [21, 22, 23]. Behavioral assessments were performed on days 0, 3, 7, 14, and 21 post‐initial PTX administration. On day 21, mouse dorsal root ganglions (DRGs) from lumbar segments L3–L5 were harvested, immediately frozen in liquid nitrogen, and stored at −80°C for subsequent analysis. The vehicle control group received i.p. injections of saline in volumes equivalent to the drug treatments according to the same schedule.

### 2.3 Behavioral Assays

#### 2.3.1 Mechanical Allodynia (von Frey Test)

Mechanical allodynia was assessed using calibrated von Frey filaments, according to established methods [24]. Mice were placed individually on an elevated wire mesh grid within a clear acrylic enclosure and allowed to acclimate for approximately 30 min. Filaments were applied perpendicularly to the plantar surface of the right hind paw, with sufficient force to cause slight bending, and held for 5 s. A positive pain response was defined as rapid paw withdrawal, shaking, or licking of the stimulated paw. Testing began with the 0.4 g filament. The Dixon ‘up‐down’ method was employed: following a positive response, the next weaker filament was applied; following a negative response, the next stronger filament was applied. This sequence started immediately after the first positive response and continued until six responses were recorded (including the initial positive). The 50% mechanical paw withdrawal threshold (PWT) was calculated from these six values using the Dixon formula.

#### 2.3.2 Thermal and Cold Hyperalgesia (Hot/Cold‐Plate Test)

Thermal and cold hyperalgesia were assessed using a hot/cold plate analgesia meter (KEW BASIS, Nanjing, China), following previously established methods [25]. Mice were brought to the testing room and allowed to acclimate in their home cages for approximately 30 min prior to testing. The temperatures for the hot and cold plates were set at 53°C ± 1°C and 4°C ± 1°C, respectively. Mice were placed gently onto the heat/cold surface, and the latency (in seconds) to the first nocifensive behavior (hind paw lick, shake, or jump) was recorded. A maximum cutoff time of 20 s was enforced to prevent tissue damage. Each mouse was tested three times, with a minimum 5‐min inter‐trial interval.

### 2.4 Single‐Nucleus RNA‐Seq Data Analysis

For the single‐nucleus RNA sequencing (snRNA‐seq) data analysis, we utilized single‐cell sequencing datasets derived from CIPN mice, which are available in the figshare repository under accession number 27627786 and have been previously described in our work [26]. Gene expression profiles were visualized using box‐and‐whisker plots, where the horizontal line within each box denotes the median value. The lower and upper bounds of the box correspond to the first (Q1) and third (Q3) quartiles, respectively, while the whiskers extend from these quartiles to the most extreme data points that fall within 1.5 times the interquartile range (IQR) of Q1 or Q3. Data points beyond this range were classified as outliers. Prior to performing group‐wise mean comparisons, the datasets were subjected to the Shapiro–Wilk test to assess normality and the Bartlett test to verify homogeneity of variance, ensuring compliance with the statistical assumptions of the subsequent analyzes.

### 2.5 Primary Culture of Mouse DRG Neurons

Isolate the DRGs from the L3‐L5 of eight 8‐week‐old C57BL/6J mice. Cut the DRGs into small pieces and rinse them with Dulbecco's Modified Eagle Medium (DMEM). Then, digest the tissue in trypsin digestion solution in a water bath at 37°C for 30 min. After digestion, terminate the process by adding DMEM and rinse the cells three times. Finally, neurons were resuspended in complete DMEM and plated onto polylysine‐coated dishes for culture.

### 2.6 Determination of Intracellular Reactive Oxygen Species (ROS) Content

Following experimental grouping and drug treatment, intracellular reactive oxygen species (ROS) levels were measured by replacing the culture medium with 1 mL of DCFH‐DA fluorescent probe diluted 1:1000 in basal medium (final concentration 10 μmol/L) and incubating at 37°C for 20 min. Cells were then washed three times with serum‐free medium before adding 1 mL of complete DMEM for observation; green fluorescence intensity was assessed using laser confocal microscopy.

### 2.7 Determination of Intracellular Mitochondrial Membrane Potential (MMP) Level

MMP was determined by adding 1 mL of serum‐free medium followed immediately by 1 mL of diluted JC‐1 staining working solution to each dish, mixing thoroughly, and incubating at 37°C for 20 min. Cells were then washed twice with JC‐1 staining buffer solution, covered with 2 mL of complete medium, and red/green fluorescence intensities were observed by confocal microscopy.

### 2.8 Measurement of Malondialdehyde (MDA) and Superoxide Dismutase (SOD) Contents in Mouse DRGs

MDA content and SOD activity were measured in DRG tissues from six mice per group. Tissues were homogenized in lysis buffer on ice, centrifuged, and the supernatant collected. MDA levels and SOD activity were then determined using commercial assay kits (Beyotime, Shanghai) according to the manufacturer's instructions.

### 2.9 Western Blot

DRGs from six mice per group were lysed in buffer containing 1% protease inhibitor and magnetic beads. After grinding, protein concentration was determined using a BCA kit. Protein samples were mixed with loading buffer, denatured at 100°C for 10 min, separated by SDS‐PAGE (80 V stacking gel, 120 V separating gel), and transferred to a PVDF membrane (400 mA, 60 min). The membrane was blocked with 5% skim milk powder for 2 h, incubated with primary antibodies (PGC‐1α, 1:5000; HO‐1, 1:2000) at 4°C overnight, then incubated with a horseradish peroxidase (HRP)‐conjugated secondary antibody (e.g., anti‐β‐actin, 1:5000) at room temperature for 2 h. Signals were detected using chemiluminescent solution and visualized with a gel imaging analyzer; band densities were quantified using ImageJ software.

### 2.10 Transmission Electron Microscopy (TEM)

DRGs from the mouse L3‐L5 were rapidly dissected and fixed in electron microscopy preservation solution at 4°C for 12 h. Samples were dehydrated through a graded ethanol series (50%, 70%, 90%, 100%; 15 min each), embedded in resin, and polymerized at ~60°C. Ultrathin sections (~100 nm) were cut, stained with uranyl acetate and lead citrate, and examined under a TEM.

### 2.11 SH‐SY5Y Cell Culture

SH‐SY5Y cells were cultured in medium supplemented with 10% FBS at 37°C in a humidified atmosphere of 5% CO_2_. The medium was replaced every 2–3 days. When cells reached 80%–90% confluence, the old medium was aspirated. Cells were gently washed twice with 2 mL PBS to remove residual medium. An appropriate volume of trypsin was added, and cells were incubated at 37°C for approximately 2 min, or until they began to detach. Trypsinization was quenched by adding an equal volume of serum‐containing medium. The cell suspension was centrifuged at 1000 rpm for 3 min. After discarding the supernatant, the cell pellet was resuspended in fresh culture medium. The desired volume of the cell suspension was then transferred to new culture vessels for continued passage.

### 2.12 TUNEL Assay

Cells were washed once with PBS and fixed in 4% paraformaldehyde for 30 min at room temperature. After PBS washing, cells were permeabilized with 0.3% Triton X‐100 in PBS for 5 min. The TUNEL reaction mixture was prepared according to the manufacturer's instructions and thoroughly mixed. Following two PBS washes, 50 μL of TUNEL reaction mixture was applied to each sample. Samples were incubated at 37°C for 60 min protected from light. After three PBS washes, nuclei were counterstained with DAPI‐containing anti‐fade mounting medium. Apoptotic cells were visualized under a fluorescence microscope (Cy3: excitation 550 nm/emission 570 nm; DAPI: excitation 358 nm/emission 461 nm).

### 2.13 Statistical Analysis

All data were analyzed using GraphPad Prism 9.5.1 software. Data meeting assumptions of normality and equal variance were compared using one‐way or two‐way repeated measures ANOVA, with Tukey's test for post hoc pairwise comparisons. Nonparametric tests were used for data violating these assumptions. Results are expressed as mean ± standard error of the mean (SEM), and a *p* value less than 0.05 was considered statistically significant.

## 3 Results

### 3.1 PACAP Dose‐Dependently Alleviates Pain Sensitivity in PIPN Mice

**FIGURE 1:**
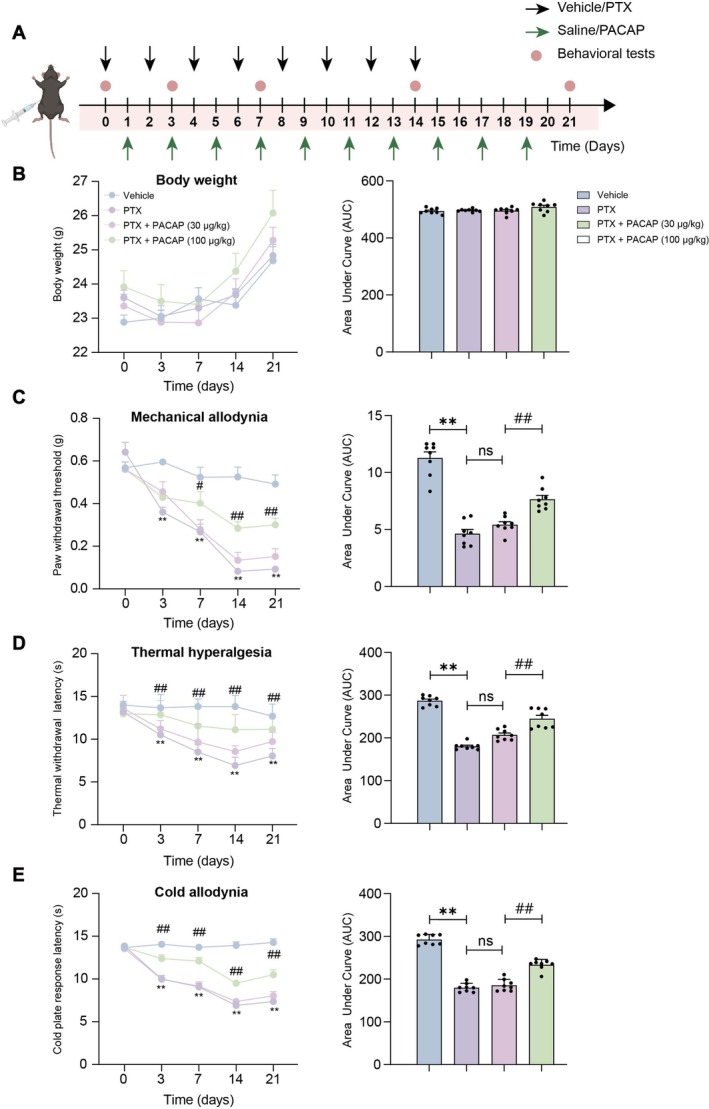
Effects of PACAP on body weight and pain‐related behaviors in mice. (A) Body weight dynamics and the corresponding area under the curve (AUC) were measured on days 1, 7, and 14 across all experimental groups. (B) Alterations in the mechanical withdrawal threshold (50% MWT) of the right hind paw, along with the AUC values, were recorded on days 1, 7, and 14. (C) Thermal withdrawal latency (TWL) changes and their AUC were evaluated on days 1, 7, and 14. (D) Cold plate response latency fluctuations and associated AUC were monitored on days 1, 7, and 14. *n* = 8; mean ± SEM; **p < 0.01 vs. Vehicle group; ^#^ *p* < 0.05, ^##^ *p* < 0.01 vs. PTX group.

As depicted in Figure 1A, the animal experimental design is illustrated schematically. To assess the impact of PACAP on body weight and analgesia in PIPN mice, we continuously monitored body weight and systematically measured pain‐related behaviors, including the 50% mechanical withdrawal threshold (MWT), tail withdrawal latency (TWL), and cold plate response latency. The results demonstrated that neither PTX nor PACAP administration had a significant effect on mouse body weight. This was further corroborated by the area under the curve (AUC) data, which showed no significant changes in body weight (*p* > 0.05, Figure 1B). Compared with the Vehicle group, PTX‐treated mice displayed significantly decreased 50% MWT (*p* < 0.01, Figure 1C), TWL (*p* < 0.01, Figure 1D), and cold plate response latency (*p* < 0.01, Figure 1E) in the right hind paw on days 3, 7, 14, and 21, thus confirming the development of PIPN. The AUC analyzes also revealed similar trends, reinforcing the consistency of these findings.

In contrast to the PTX group, mice treated with the combination of PTX + PACAP (30 μg/kg) did not exhibit significant improvements in pain measures. However, mice administered the higher dose of PACAP (100 μg/kg) showed a notable increase in 50% MWT (*p* < 0.05, Figure 1C), TWL (*p* < 0.05, Figure 1D), and cold plate response latency (*p* < 0.01, Figure 1E) on days 7 and 14. Consistent with previous results, the AUC analyzes mirrored these trends. Collectively, these findings indicate that a high dose of PACAP exerts a significant analgesic effect in PIPN mice, providing strong evidence for a dose‐dependent mechanism underlying pain relief.

### 3.2 PACAP Restores PTX‐Induced Increases in ROS and Mitochondrial Membrane Potential Changes in Primary Mouse DRG Neurons

**FIGURE 2:**
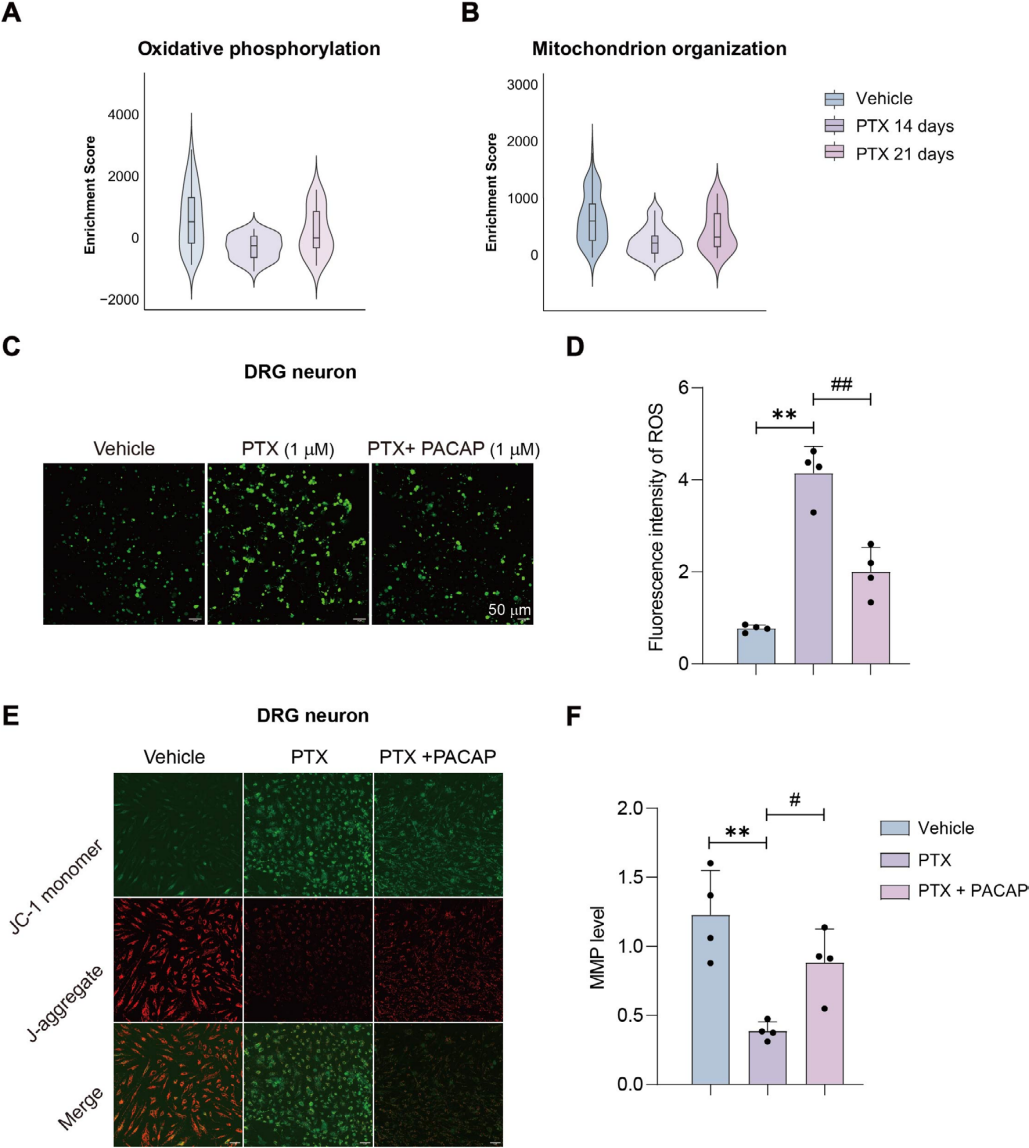
ROS levels and mitochondrial membrane potential (MMP) in primary DRG neurons. (A, B) Suppressed oxidative phosphorylation (A) and disrupted mitochondrial organization (B) in C_LTMR neurons of DRG at 14 and 21 days post‐paclitaxel treatment from sn‐RNAseq analysis. (C) Representative fluorescence images (20×) of ROS production in primary mouse DRG neurons; scale bar = 100 μm. (D) Quantification of ROS levels normalized to the Vehicle group. (E) Representative fluorescence images (20×) of MMP (JC‐1 staining) in DRG neurons; scale bar = 100 μm. Red: JC‐1 aggregates (high MMP); green: JC‐1 monomers (low MMP). MMP was calculated as the red/green fluorescence ratio. (F) Quantification of MMP levels across groups. *n* = 5, mean ± SEM. **p < 0.01 vs. vehicle group; ^##^ *p* < 0.01 vs. PTX group.

To further investigate the regulatory effects of PACAP on PIPN mice, we re‐analyzed our previous single‐nuclear RNA sequencing (sn‐RNAseq) data from the PIPN mouse model. As shown in Figure 2A,B, the snRNA‐seq data revealed that type C low‐threshold mechanoreceptors (C_LTMR) in DRG are key mediators of paclitaxel‐induced pain hypersensitivity in mice. Moreover, C_LTMR neurons in the DRG exhibited suppressed oxidative phosphorylation and mitochondrial organization at both 14 and 21 days post‐paclitaxel treatment (Figure 2A,B). These results suggest that mitochondrial dysfunction in DRG neurons may be a key feature of PIPN. To explore this further, we examined the ROS and MMP levels in PTX‐treated primary DRG neurons; we quantified intracellular ROS levels and MMP using commercial assay kits. Results revealed that PTX significantly increased ROS production compared to the vehicle control group (*p* < 0.01; Figure 2C,D). Conversely, co‐treatment with PTX and PACAP (PTX + PACAP) markedly reduced ROS levels relative to the PTX group (*p* < 0.01; Figure 2C,D).

For MMP assessment, we employed the JC‐1 fluorescence probe. This cationic dye forms red fluorescent aggregates at high MMP but shifts to green fluorescent monomers when MMP declines; thus, the red/green fluorescence ratio reflects MMP status. The results indicated that, compared with the vehicle group, MMP was significantly reduced in the PTX group (*p* < 0.01; Figure 2E,F). Furthermore, PTX + PACAP co‐treatment significantly restored MMP compared to PTX alone (*p* < 0.01; Figure 2E,F).

### 3.3 PACAP Upregulates PGC‐1α and HO‐1 Expression and Attenuates Oxidative Stress in the DRG of PIPN Mice

**FIGURE 3:**
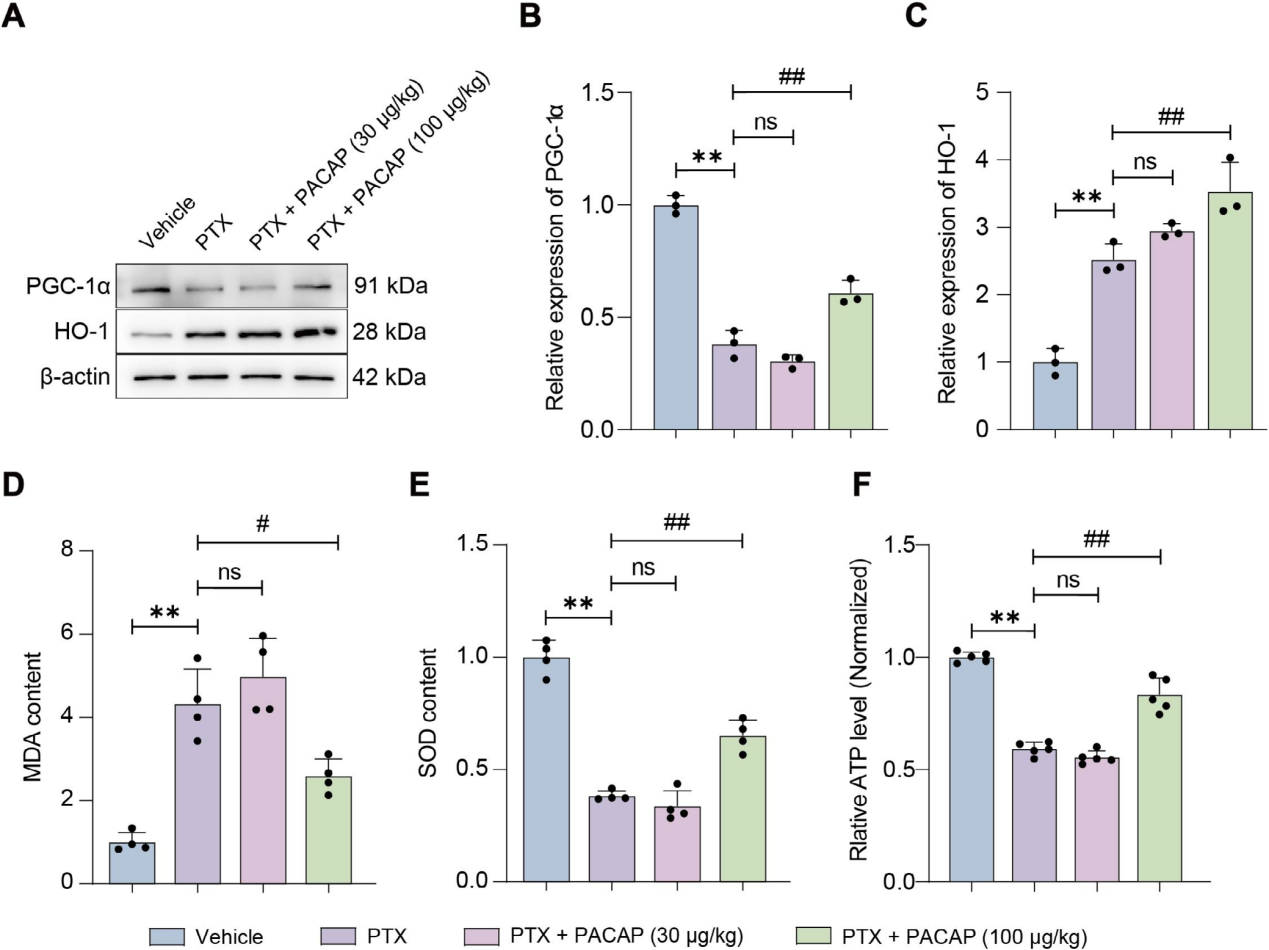
Oxidative Stress and Protein Expression of PGC‐1α and HO‐1 in the DRG of PIPN mice. (A) Representative Western blot images showing PGC‐1α and HO‐1 protein expression in DRG tissues across different groups. (B) Quantitative analysis of PGC‐1α protein levels normalized to the vehicle group. (C) Quantitative analysis of HO‐1 protein levels normalized to the vehicle group. (D) Statistical analysis of MDA content in DRG tissues, normalized to the vehicle group. (E) Statistical analysis of SOD activity in DRG tissues, normalized to the vehicle group. (F) The adenosine triphosphate (ATP) levels in the DRG from PIPN mice. *n* = 6, mean ± SEM. **p < 0.01 vs. vehicle group; ^##^ *p* < 0.01 vs. PTX group.

To further explore the potential mechanisms by which PACAP alleviates PIPN in mice, we analyzed protein expression in DRG tissues. Western blot results revealed that, compared to the Vehicle group, the PTX group exhibited significantly reduced PGC‐1α expression (*p* < 0.01; Figure 3A,B) but markedly increased heme oxygenase‐1 (HO‐1) levels (*p* < 0.01; Figure 3A,C). This HO‐1 upregulation likely represents a compensatory antioxidant response to PTX‐induced oxidative stress. Compared to the PTX group, the PTX + PACAP (30 μg/kg) group showed no significant changes in PGC‐1α or HO‐1 expression. However, the PTX + PACAP (100 μg/kg) group demonstrated significant upregulation of both PGC‐1α (*p* < 0.01; Figure 3A,B) and HO‐1 (*p* < 0.01; Figure 3A,C). These findings suggest that high‐dose PACAP promotes mitochondrial function recovery and enhances antioxidant capacity by inducing PGC‐1α and HO‐1 expression, potentially protecting DRG neurons and alleviating PTX‐induced pain.

To assess the effect of PACAP on oxidative stress, we measured MDA content and SOD activity in DRG. Compared with the Vehicle group, the PTX group exhibited significantly elevated MDA levels (*p* < 0.01; Figure 3D) and decreased SOD activity (*p* < 0.01; Figure 3E). The PTX + PACAP (30 μg/kg) group showed no significant changes in MDA or SOD. Conversely, the PTX + PACAP (100 μg/kg) group showed a significant decrease in MDA content (*p* < 0.01; Figure 3D) and an increase in SOD activity (*p* < 0.01; Figure 3E). Additionally, we measured the adenosine triphosphate (ATP) levels in the DRG to assess mitochondrial functions in PIPN mice. The data showed that PTX significantly decreased the ATP levels in the DRG. The PTX + PACAP (30 μg/kg) group did not show significant changes in ATP, whereas the PTX + PACAP (100 μg/kg) group exhibited a significant increase in ATP levels (*p* < 0.01; Figure 3F). These results indicate that high‐dose PACAP effectively attenuates oxidative stress and improves mitochondrial functions in the DRG of PIPN mice.

### 3.4 PACAP Attenuates Mitochondrial Damage in the DRG of PIPN Mice

**FIGURE 4:**
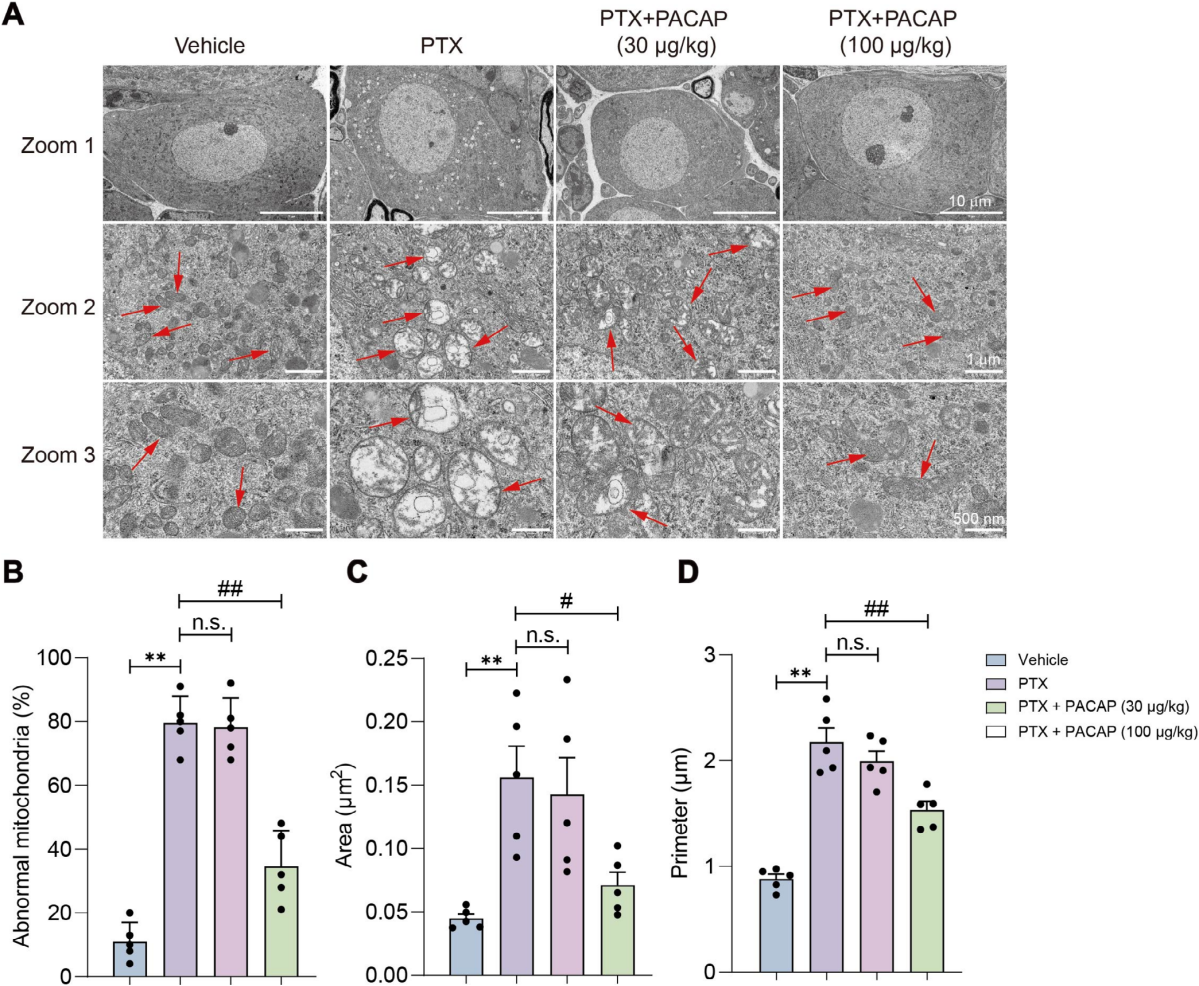
Mitochondrial ultrastructure in DRG neurons of PIPN mice. (A) Ultrastructural images showing the morphology of mitochondria in DRG at 12,000× magnification; scale bar = 500 nm. The red arrows indicate mitochondria, allowing clear visualization of their shape, membrane integrity, and cristae structure across different experimental groups. (B–D) Quantitative analysis of the ratios of abnormal mitochondria (B), mitochondrial area (C), and mitochondrial perimeter (D). *n* = 5, mean ± SEM. **p < 0.01 vs. vehicle group; ^#^ *p* < 0.05, ^##^ *p* < 0.01 vs. PTX group.

To investigate the impact of PACAP on mitochondrial morphology in the DRG of PIPN mice, we examined ultrastructural changes using transmission electron microscopy (TEM) and performed quantitative morphometric analysis. TEM revealed intact mitochondria in the Vehicle group, displaying an oval shape, continuous double membranes, and well‐organized cristae (Figure 4A). In contrast, the PTX group exhibited severe mitochondrial damage, characterized by widespread vacuolation, outer membrane rupture, loss of structural integrity, and cristae that were fused, disorganized, or absent (Figure 4A). Quantitative analysis showed that the PTX + PACAP (30 μg/kg) group exhibited no significant improvement versus the PTX group in the ratio of abnormal mitochondria, mitochondrial area, or mitochondrial perimeter; however, the PTX + PACAP (100 μg/kg) group demonstrated significantly reduced ratios of abnormal mitochondria (*p* < 0.01; Figure 4B), decreased mitochondrial area (*p* < 0.01; Figure 4C), and diminished mitochondrial perimeter (*p* < 0.01; Figure 4D). These findings indicate that high‐dose PACAP (100 μg/kg) significantly mitigates PTX‐induced mitochondrial damage in DRG neurons, preserving mitochondrial integrity.

### 3.5 PACAP Upregulates PGC‐1α and HO‐1 Expression in SH‐SY5Y Cells as Well

**FIGURE 5:**
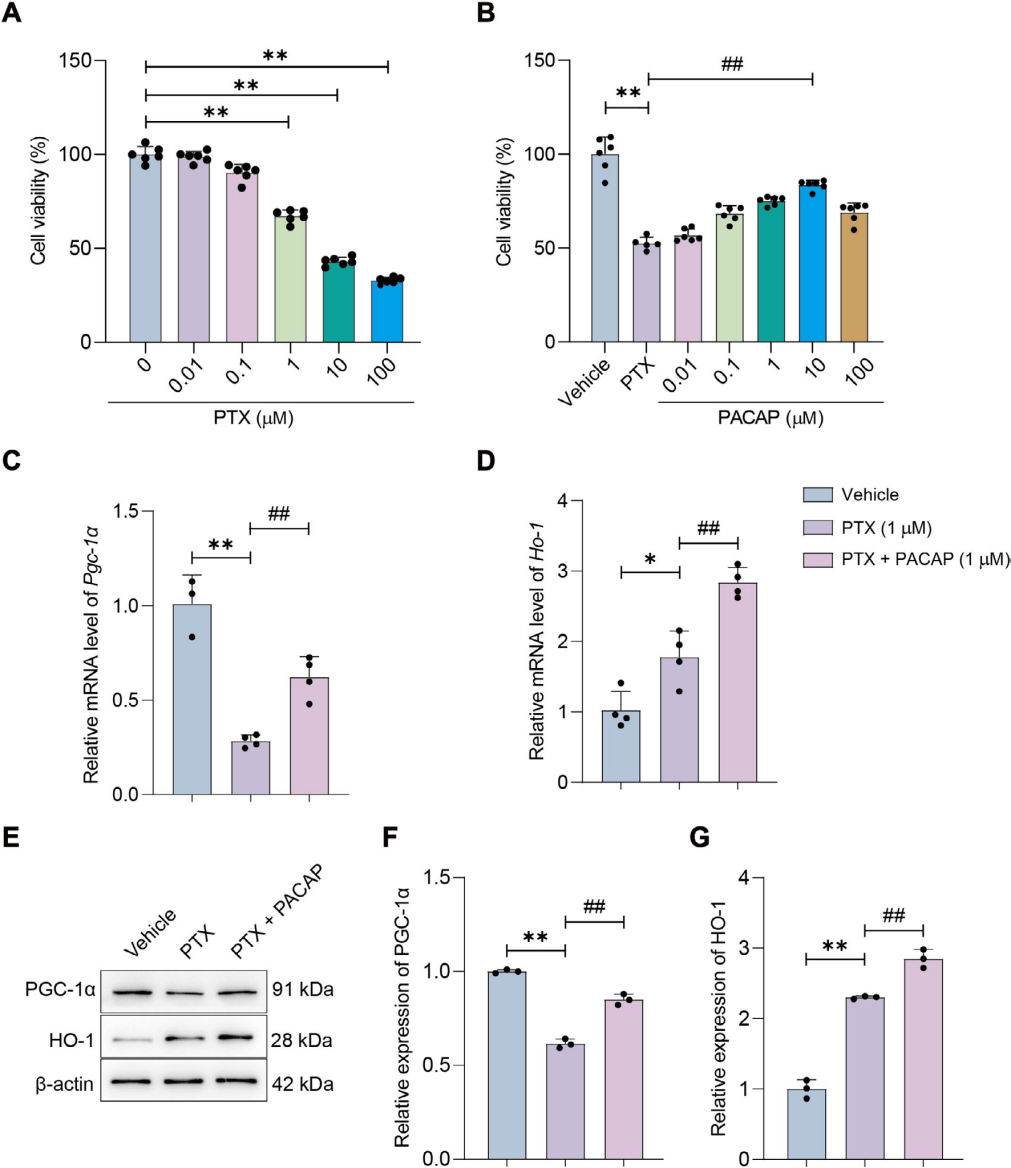
Effects of PACAP on PTX‐induced cytotoxicity, mitochondrial markers, and stress responses in SH‐SY5Y cells. (A) Viability of SH‐SY5Y cells treated with increasing PTX concentrations (MTT assay). (B) Viability of SH‐SY5Y cells pretreated with PACAP followed by PTX (MTT assay). Mean ± SEM. *n* = 6. **p < 0.01 vs. Control group; ^#^ *p* < 0.01, ^##^ *p* < 0.01 vs. PTX group. (C, D) Quantitative RT‐PCR analysis of the mRNA expression levels of *Pgc‐1α* (C) and *Ho‐1* (D) in each group of SH‐SY5Y cells. Mean ± SEM. *n* = 4. **p < 0.01 vs. Control group; ^##^ *p* < 0.01 vs. PTX group. (E) Western blot analysis of PGC‐1α, HO‐1 protein expression levels in each group of SH‐SY5Y cells. (F, G) Semi‐quantitative analysis of PGC‐1α (F) and HO‐1 (G) protein levels. Mean ± SEM. *n* = 3. **p < 0.01 vs. Control group; ^##^ *p* < 0.01 vs. PTX group.

To further investigate the effect of PACAP on mitochondrial protection, we used SH‐SY5Y cells. Dose–response analysis showed PTX (0.01–100 μmol/L, 24 h) reduced cell viability dose‐dependently (Figure 5A). While 0.1 μmol/L PTX caused minimal damage (90% survival), concentrations > 10 μmol/L exhibited excessive cytotoxicity. PTX 1 μmol/L reduced viability to ~40% (*p* < 0.01 vs. control; Figure 5A) and was selected for modeling. Pretreatment with PACAP (0.1–100 μmol/L, 2 h) concentration‐dependently attenuated PTX‐induced cytotoxicity. PACAP 1 μmol/L provided maximal protection (*p* < 0.01 vs. PTX group; Figure 5B) and was used for subsequent assays.

We then investigated the effect of PACAP on PTX‐induced changes in PGC‐1α and HO‐1 expression. PTX treatment significantly decreased *PGC‐1α* mRNA levels, suggesting suppressed mitochondrial biogenesis and antioxidant defenses, while increasing *HO‐1* mRNA expression, indicating compensatory stress responses (*p* < 0.01 vs. control; Figure 5C,D). Co‐treatment with PACAP (1 μmol/L) significantly elevated *PGC‐1α* and *HO‐1* mRNA levels compared to PTX alone (*p* < 0.01 vs. PTX group; Figure 5C,D). Consistent with mRNA data, PTX reduced PGC‐1α protein but increased HO‐1 (*p* < 0.01 vs. control; Figure 5E–G), while co‐treatment with PACAP significantly upregulated both proteins (*p* < 0.01 vs. PTX group; Figure 5E–G). These findings suggest that PACAP promotes mitochondrial function and antioxidant capacity while strengthening stress‐protective responses, offering comprehensive protection against PTX‐induced oxidative damage.

### 3.6 PACAP Restores PTX‐Induced Increases in ROS, Mitochondrial Membrane Potential Changes, and Cell Apoptosis in SH‐SY5Y Cells as Well

**FIGURE 6:**
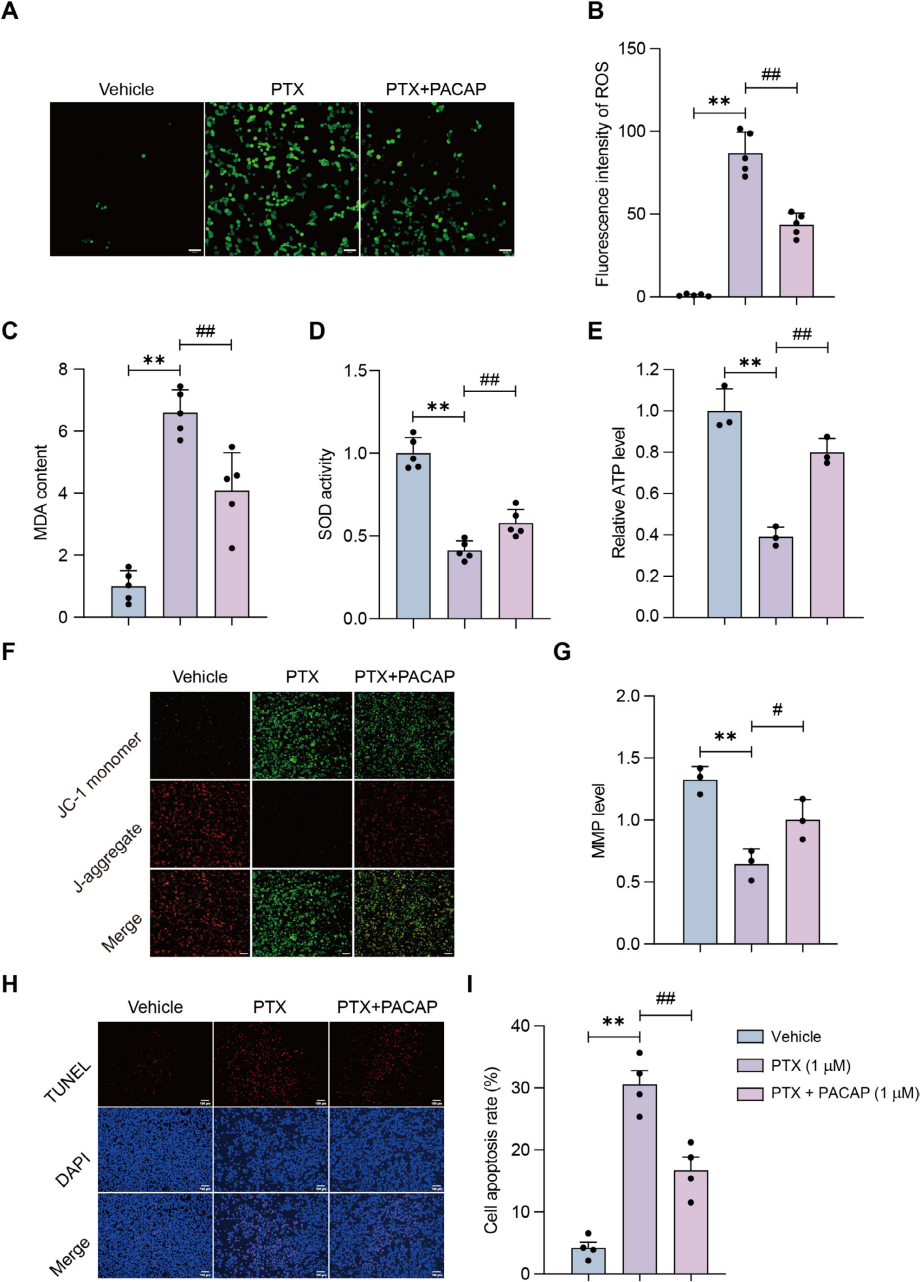
Effects of PACAP on PTX‐induced increases in ROS, mitochondrial membrane potential changes, and cell apoptosis in SH‐SY5Y cells. (A) Representative images (20×) of intracellular ROS (DCFH‐DA staining; green fluorescence); scale bar = 100 μm. (B) Quantification of ROS fluorescence intensity. (C) Malondialdehyde (MDA) content. (D) Superoxide dismutase (SOD) activity. (E) The ATP levels in SH‐SY5Y cells. Mean ± SEM. *n* = 5. **p < 0.01 vs. Control group; ^##^ *p* < 0.01 vs. PTX group. (F) Representative images (20×) of MMP (JC‐1 staining); scale bar = 100 μm. (G) Quantification of MMP (red/green fluorescence ratio). (H) Representative images (20×) of TUNEL staining; scale bar = 100 μm. (I) Quantification of cell apoptosis based on TUNEL signals. Mean ± SEM. *n* = 3–5. **p < 0.01 vs. Control group; ^##^ *p* < 0.01 vs. PTX group.

To further investigate the regulatory effects of PACAP on mitochondrial protection, we measured ROS levels, MMP, and cell apoptosis in PTX‐treated SH‐SY5Y cells. Results showed PTX significantly increased ROS production compared to the vehicle control group (*p* < 0.01; Figure 6A,B). In contrast, co‐treatment with PTX and PACAP (PTX + PACAP) markedly reduced ROS levels relative to the PTX group (*p* < 0.01). Compared to the Vehicle group, the PTX group exhibited elevated MDA levels (*p* < 0.01; Figure 6C) and decreased SOD activity (*p* < 0.01; Figure 6D). PACAP treatment, however, significantly decreased MDA content (*p* < 0.01) and increased SOD activity (*p* < 0.01). Elevated ROS levels indicate heightened oxidative stress, while increased MDA reflects enhanced lipid peroxidation, suggesting severe damage to cell membranes and other biological structures. The declined SOD activity weakens the antioxidant defense system of cells, reducing its ability to combat oxidative stress. Additionally, PTX reduced ATP levels in SH‐SY5Y cells, while PACAP treatment restored ATP levels (*p* < 0.01; Figure 6E). As a critical indicator of mitochondrial health, MMP was analyzed to assess the role of PGC‐1α in mitochondrial function regulation. MMP analysis showed PTX significantly decreased MMP compared to the vehicle group, whereas PACAP co‐treatment significantly restored MMP (*p* < 0.01; Figure 6F,G). Given that mitochondria are the energy hub of cells and MMP reflects their functional status, this decrease demonstrates notable mitochondrial impairment. Furthermore, we investigated the effect of PACAP on cell apoptosis. As shown in Figure 6H,I, TUNEL staining results revealed that the apoptotic signal was significantly higher in the PTX‐treated group but lower in the PTX + PACAP group (*p* < 0.05; Figure 6H,I).

### 3.7 PGC‐1α Is Essential for PACAP‐Mediated Protection Against PTX‐Induced Oxidative Stress and Mitochondrial Damage in SH‐SY5Y Cells

**FIGURE 7:**
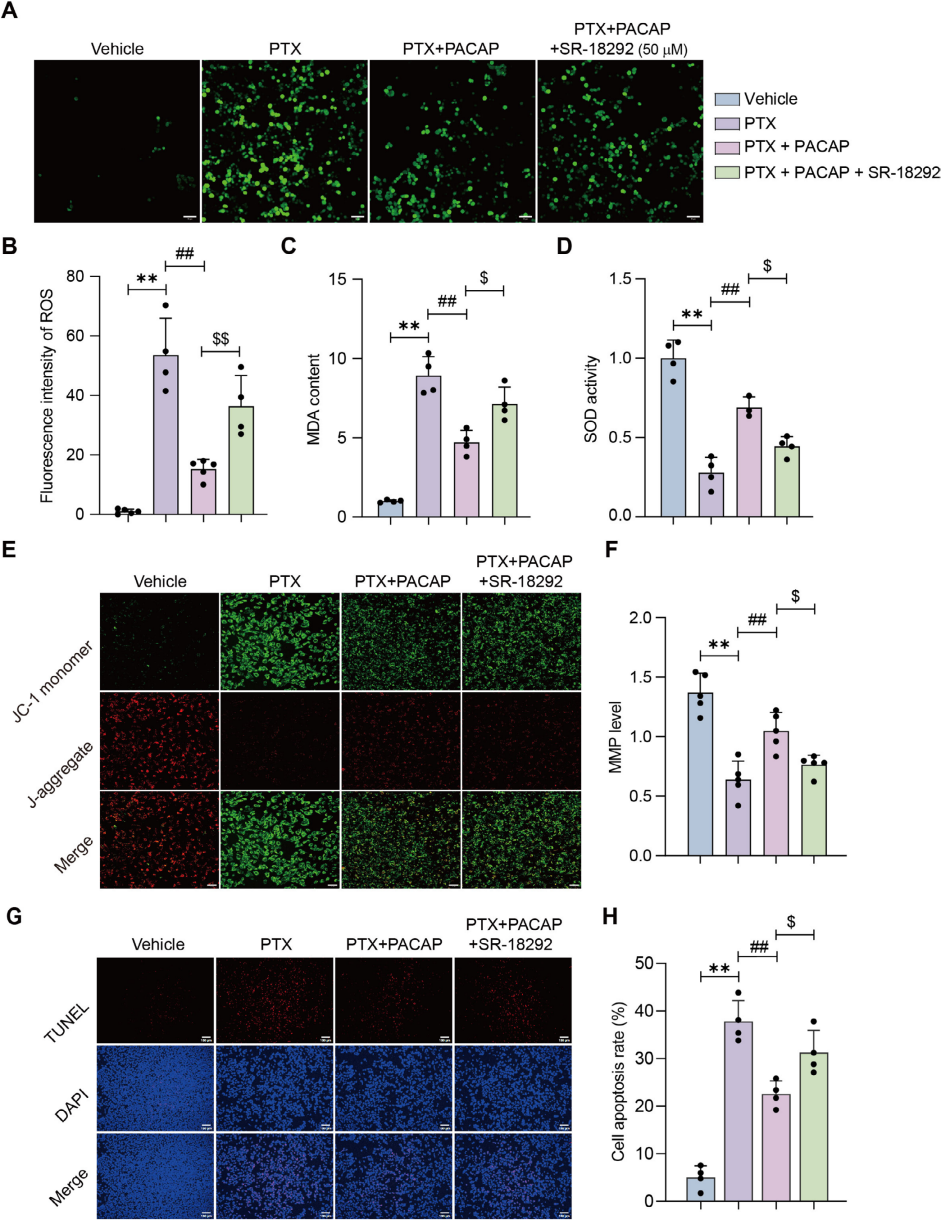
Role of PGC‐1α in mediating PACAP protection against PTX‐induced oxidative stress and mitochondrial dysfunction in SH‐SY5Y cells. Cells were treated with the PGC‐1α inhibitor SR‐18292. (A) Representative images (20×) of intracellular ROS (DCFH‐DA staining; green fluorescence); scale bar = 100 μm. (B) Quantification of ROS fluorescence intensity. (C) Malondialdehyde (MDA) content. (D) Superoxide dismutase (SOD) activity. Mean ± SEM. *n* = 4. **p < 0.01 vs. Control group; ^##^ *p* < 0.01 vs. PTX group; ^$^ *p* < 0.05, ^$$^ *p* < 0.01 vs. PTX + PACAP group. (E) Representative images (20×) of MMP (JC‐1 staining); scale bar = 100 μm. (F) Quantification of MMP (red/green fluorescence ratio). (G) Representative images (20×) of TUNEL staining; scale bar = 100 μm. (H) Quantification of cell apoptosis based on TUNEL signals. Mean ± SEM. *n* = 5. **p < 0.01 vs. Control group; ^##^ *p* < 0.01 vs. PTX group; ^$^ *p* < 0.05 vs. PTX + PACAP group.

To elucidate the role of PGC‐1α in PACAP‐mediated protection against PTX‐induced mitochondrial damage, we employed the PGC‐1α inhibitor SR‐18292 and analyzed oxidative stress markers and mitochondrial function in SH‐SY5Y cells. As depicted in Figure 7, SR‐18292 significantly reversed the effect of PACAP on intracellular ROS levels (*p* < 0.01; Figure 7A,B) and MDA content (*p* < 0.05; Figure 7C) in SH‐SY5Y cells. Concurrently, the activity of SOD significantly decreased in the SR‐18292 treatment group (*p* < 0.05; Figure 7D). These results indicate that SR‐18292 reverses the protective effect of PACAP against oxidative damage, shifting the intracellular redox balance towards oxidation.

We further measured mitochondrial membrane potential (MMP) and found that the SR‐18292 treatment group exhibited a significantly lower MMP compared to the PTX + PACAP group (*p* < 0.05; Figure 7E,F). Additionally, SR‐18292 significantly reversed the effect of PACAP on TUNEL staining intensity (*p* < 0.01; Figure 7G,H). Collectively, these findings demonstrate that inhibition of PGC‐1α by SR‐18292 impairs both antioxidant capacity and mitochondrial function in SH‐SY5Y cells. By enhancing oxidative stress, reducing antioxidant enzyme activity, and decreasing MMP, SR‐18292 abolishes the protective effects of PACAP against PTX‐induced injury. Additionally, the data reveal that this inhibition also leads to an apoptotic imbalance, as evidenced by the significant reversal of the effect of PACAP on the TUNEL signal. These results highlight the crucial role of PGC‐1α in mediating the neuroprotective effect of PACAP, underscoring its importance in maintaining cellular homeostasis, including redox balance, mitochondrial integrity, and apoptotic regulation, during PTX exposure.

### 3.8 PACAP Does Not Affect the Anti‐Cancer Effect of PTX Both In Vitro and In Vivo

**FIGURE 8:**
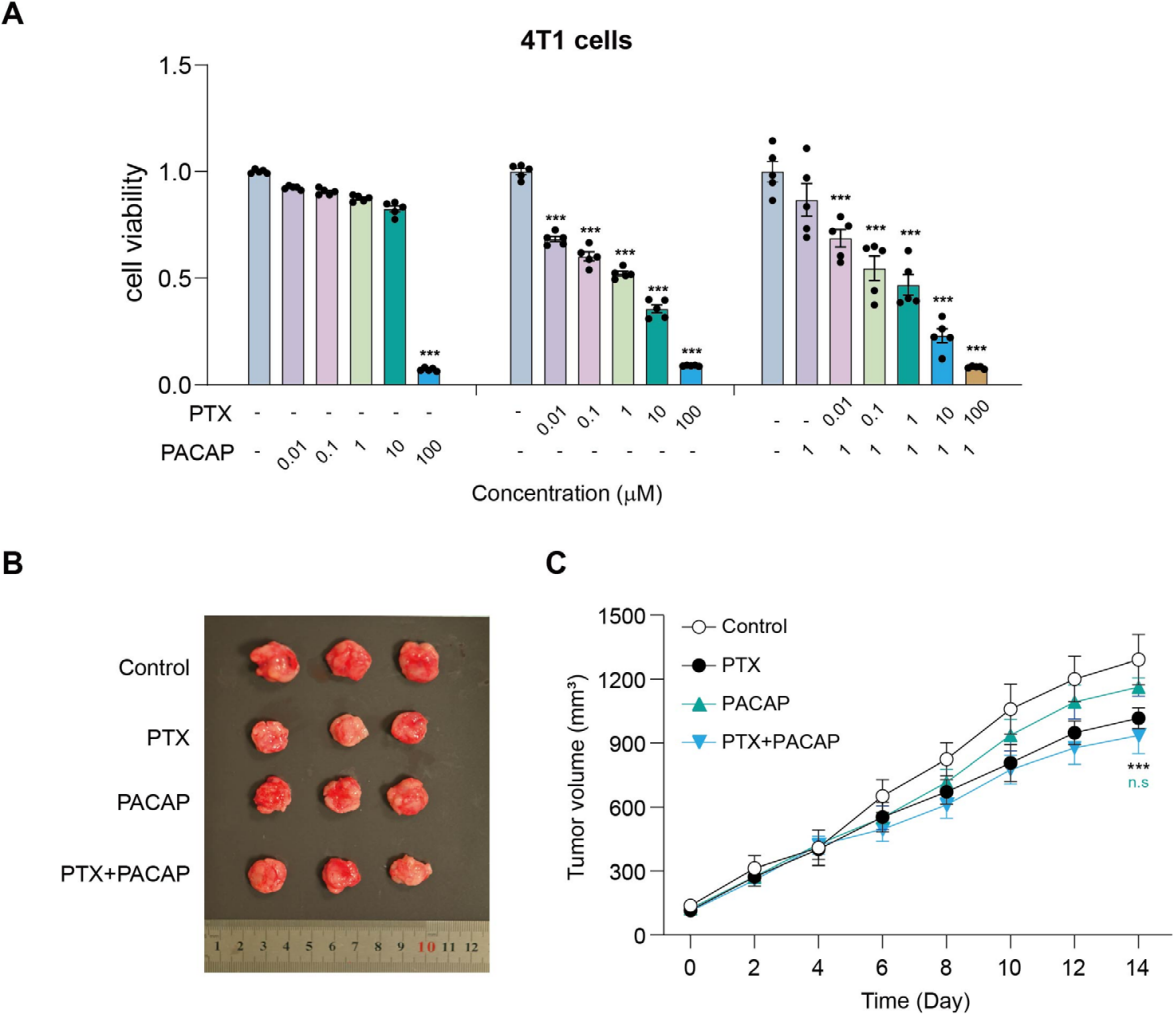
The effect of PACAP on the anti‐tumor efficacy of PTX. (A) In vitro cell viability assay using human breast cancer cells (4T1) treated with PACAP and PTX. (B) Representative images of tumor tissues from an in vivo tumor growth study in a mouse xenograft model. (C) Statistical analysis of tumor volume in each xenograft mouse. Mean ± SEM. *n* = 6. ***p < 0.001 vs. Control group.

To assess the impact of PACAP on the anti‐cancer effect of PTX, we conducted cell viability assays and tumor‐bearing mouse model experiments. PTX significantly reduced the viability of cultured human breast cancer 4 T1 cells in a dose‐dependent manner in vitro (*p* > 0.05; Figure 8A). Notably, the addition of PACAP did not modify the anti‐tumor effect of PTX on these cells (*p* > 0.05; Figure 8A), indicating that PACAP did not interfere with the cytotoxicity of PTX in the cell culture setting. In the in vivo experiments using tumor‐bearing mice, PTX treatment led to a significant reduction in tumor volume (*p* < 0.01; Figure 8B,C). Meanwhile, co‐administration of PACAP did not diminish the anti‐tumor efficacy of PTX (*p* > 0.05; Figure 8B,C). This result further confirmed that PACAP does not compromise PTX's therapeutic action against tumors in a living organism. Collectively, these findings suggest that PACAP is a promising endogenous peptide for combination with PTX. As it does not affect the anti‐cancer effect of PTX either in vitro or in vivo, PACAP could potentially be co‐administered with PTX to prevent and manage PIPN without interfering with the anti‐tumor benefits.

## 4 Discussion

This study provides compelling evidence that PACAP can effectively reduce peripheral neuropathy caused by PTX in mice. The primary mechanism involves activation of the PGC‐1α pathway, which helps improve mitochondrial function and reduce oxidative stress within DRG. Our behavioral data show that a high dose of PACAP (100 μg/kg) significantly reversed key symptoms of neuropathy, including mechanical allodynia, thermal hyperalgesia, and cold hypersensitivity. Interestingly, this effect was dose‐dependent; the lower dose (30 μg/kg) was ineffective, suggesting that a certain threshold of PACAP is necessary to see these benefits. Importantly, PACAP did not interfere with the anti‐cancer effect of PTX both in vitro and in vivo, indicating it could potentially serve as a safe adjunct treatment to address chemotherapy‐induced neuropathy without compromising cancer therapy.

PTX is a commonly used anticancer drug, but its induced PIPN seriously affects patients' quality of life. The main symptoms of PIPN include mechanical, thermal, and cold allodynia. Its pathogenesis is complex, involving oxidative stress, mitochondrial dysfunction, neuroinflammation, and other aspects [5, 27]. Oxidative stress refers to the imbalance between ROS and antioxidants in the body, leading to excessive accumulation of ROS in cells and tissues, thereby damaging cellular structure and function [28]. PACAP targets two interconnected pathological hallmarks of PIPN: oxidative stress and mitochondrial dysfunction [29]. Our data reveal that PTX triggers ROS overload in DRG neurons, depletes SOD activity, elevates lipid peroxidation (MDA), and collapses MMP—all signatures of profound cellular distress. MDA is one of the end products of lipid peroxidation and is often used as an indicator to evaluate cellular oxidative damage. SOD is a class of important antioxidant enzymes that catalyze the dismutation of superoxide anions into hydrogen peroxide and oxygen, thereby reducing free radical damage to cells. Both are commonly used to assess the oxidative stress status of the body [30]. This study found that the content of MDA in DRG tissues of mice in the PTX group was increased, while SOD activity was decreased, indicating that PTX induced significant oxidative stress.

Mitochondria are core organelles of cellular energy metabolism and oxidative stress, and their dysfunction is closely related to neuropathic pain [31]. It has been reported that PACAP can maintain mitochondrial function, reduce oxidative stress and apoptosis [32], and improve mitochondrial respiratory chain efficiency and increase ATP synthesis [33]. This study found that the MMP level in primary DRG cells treated with PTX was significantly decreased, while PACAP partially restored MMP. Additionally, electron microscopy observations revealed a significant increase in abnormally shaped mitochondria in DRG tissues of PTX group mice, while high‐dose PACAP treatment improved mitochondrial morphology. PACAP administration not only normalizes these biomarkers but also rescues mitochondrial ultrastructure, reversing cristae disintegration and vacuolation observed via electron microscopy. This result is consistent with previous studies showing that PACAP has antioxidant effects [33], further confirming the protective effect of PACAP on neurons. This dual‐action positions PACAP uniquely among PIPN therapeutics by addressing root pathological mechanisms rather than merely masking symptoms.

Although there is limited literature on PACAP regulating PGC‐1α, this study reveals that high‐dose PACAP modulates mitochondrial function by enhancing PGC‐1α expression. As a crucial regulator of mitochondrial biogenesis, increased PGC‐1α expression promotes the generation and functional restoration of mitochondria, thereby alleviating oxidative stress injury [34]. In the context of neuropathic pain, the down‐regulation of PGC‐1α expression is usually closely related to mitochondrial dysfunction, heightened oxidative stress, and neuronal injury [35]. Diving deeper into the mechanisms, our data highlight PGC‐1α as a central player. PGC‐1α is a master regulator of mitochondrial biogenesis and antioxidant defenses. PTX alone suppressed PGC‐1α expression in the DRG, which aligns with the observed mitochondrial dysfunction and oxidative stress. Conversely, high‐dose PACAP significantly increased PGC‐1α levels. Using a PGC‐1α inhibitor, SR‐18292, we confirmed that blocking this pathway abolished the protective effects of PACAP, linking PGC‐1α activation directly to its neuroprotective and pain‐relieving functions.

Furthermore, this study found that high‐dose PACAP increased HO‐1 protein expression. We speculate that PACAP enhances the antioxidant stress capacity by increasing HO‐1 protein expression, further reducing nerve cell injury. HO‐1 is a stress response protein with antioxidant and anti‐inflammatory effects. In neuropathic pain models, increased HO‐1 expression has been confirmed to significantly alleviate pain hypersensitivity [36], and its upregulation may be a compensatory response of the body to oxidative stress. Notably, we identify a novel synergistic dimension: PACAP concurrently upregulates HO‐1, amplifying endogenous antioxidant responses beyond PGC‐1α‐mediated pathways. While HO‐1 induction represents a known compensatory stress response, PACAP's potentiation of this mechanism—potentially via Nrf2 crosstalk—creates a dual‐defense network that simultaneously rebuilds mitochondrial capacity (via PGC‐1α) and neutralizes free radicals (via HO‐1/SOD). This coordinated transcriptional reprogramming distinguishes PACAP from single‐target therapies. This dual action likely provides a more robust defense, helping neurons recover and reducing pain symptoms. In summary, PACAP appears to initiate a cascade whereby it activates PGC‐1α, which in turn promotes mitochondrial biogenesis and function, and enhances antioxidant capacity. It also induces HO‐1, adding an extra layer of protection. Together, these effects help curb the neuronal damage caused by PTX and alleviate neuropathic pain.

However, several questions remain. It will be important to clarify how PACAP activates PGC‐1α at the molecular level—specifically, through which receptors and signaling pathways (e.g., cAMP/PKA, MAPK), and to identify the downstream genes responsible for mitochondrial and antioxidant improvements. Understanding how PACAP influences HO‐1 regulation is another key area for future research. From the translational perspective, exploring alternative delivery methods, such as intrathecal or localized injections, could optimize PACAP's efficacy and safety profile. Additionally, investigating the long‐term effects and efficacy in models that mimic chronic PIPN will be crucial for clinical relevance. PACAP and its receptors are widely distributed in the body [37, 38]. Future studies need to further elucidate the precise mechanisms of action of high‐dose PACAP on different systems of the body under various disease and physiological states, so that better balance its neurotherapeutic benefits and potential risks to other systems.

In conclusion, PACAP significantly increased the 50% MWT, prolonged the TWL and cold pain response latency in PIPN mice, reduced ROS content in primary DRG cells, improved MMP levels, protected mitochondrial morphology in DRG tissues, and increased the protein expression of PGC‐1α and HO‐1, thereby inhibiting oxidative stress, protecting mitochondria, and effectively alleviating PTX‐induced PIPN in mice. This study is the first to systematically investigate the role of PACAP in PTX‐induced PIPN in mice and reveals a new mechanism by which PACAP alleviates oxidative stress and mitochondrial damage in DRG of PIPN mice by upregulating PGC‐1α and HO‐1 proteins, thereby relieving chemotherapy‐induced neuropathy. This provides a new direction for improving the quality of life of cancer patients and developing chemotherapeutic drugs with reduced adverse reactions, helps to deepen the understanding of the mechanism of chemotherapy‐induced neuropathy, and provides a theoretical basis for the development of new treatment strategies. Importantly, PACAP did not compromise the anti‐tumor effects of PTX, making it an attractive candidate for further development to improve quality of life for cancer patients undergoing chemotherapy.

## Author Contributions

W.S. and M.Z. were responsible for the conceptualization and design of the study, data analysis, interpretation, and manuscript writing. R.M., C.W., H.H., S.S., Z.L., D.C., and H.Y. conducted animal experiments; W.S., M.Z., R.M., C.W., H.H., S.S., Z.L., D.C., and H.Y. reviewed the manuscript. W.S. and M.Z. obtained financial support from grants.

## Funding

This work was supported by the National Natural Science Foundation of China (Grants 82171378, 82401438), Science, Technology and Innovation Commission of Shenzhen Municipality (Grants JCYJ20240813114512016, JCYJ20240813152049062), Shenzhen Nanshan District Healthcare System Science and Technology Key Projects (Grant NSZD2023003) and Medical‐Engineering Interdisciplinary Research Foundation of Shenzhen University (Grant 2023YG031).

## Ethics Statement

All experiment procedures in this study were approved by the Institutional Animal Care and Use Committee (IACUC) of Shenzhen Lingfutuopu Biotechnology Co. Ltd. (Ethics Approval No. TOP‐IACUC‐2024‐0106).

## Consent

The paper has been read and approved by all authors. All authors approved the submission of this paper for publication. All authors confirmed that neither the manuscript submitted nor any part of it has been published or is being considered for publication elsewhere.

## Conflicts of Interest

The authors declare no conflicts of interest.

## Data Availability

The data that support the findings of this study are available from the corresponding author upon reasonable request.
